# Editorial: Advances in robots for learning

**DOI:** 10.3389/frobt.2025.1584122

**Published:** 2025-04-04

**Authors:** Daniel Tozadore, Jauwairia Nasir, Wafa Johal, Michelle M. Neumann

**Affiliations:** ^1^ CHILI Lab, School of Information and Computer Science, École Polytechnique Fédérale de Lausanne (EPFL), Lausanne, Switzerland; ^2^ Chair of Human-Centered Artificial Intelligence, University of Augsburg, Augsburg, Germany; ^3^ School of Computing and Information Systems, University of Melbourne, Parkville, VIC, Australia; ^4^ Faculty of Education, Southern Cross University, Gold Coast, QLD, Australia

**Keywords:** robots for learning, human robot ineraction, education, artificial intelligence, social robots

## 1 Introduction

Robots are increasingly demonstrating significant potential as learning and teaching companions for children in classrooms or at home, for elderly people to help maintain their cognitive and physical abilities, and for learners with deficiencies by facilitating personalized instruction by adapting content to their specific needs. Robots show the potential to improve individual adaptation by learning from and with the user.

Currently, several research projects aim to apply HRI to education and learning across a broader range of disciplines beyond STEM, such as language and handwriting. These projects also extend beyond imparting domain-specific skills, like computational thinking, to fostering collaborative skills and addressing diverse end-users, including children (neurotypical and neurodivergent), adults, and the elderly (both healthy and cognitively challenged).

Since the first Research Topic in this series explored how HRI can push the boundaries of education in various ways, a follow-up Research Topic would be valuable in understanding how advancements in the field are progressing, particularly in light of the rapid growth of AI over the past decade.

Therefore, the current Research Topic highlights advancements in social robotics, particularly exploring innovative algorithms and computational models within learning environments. Emphasis was placed on contributions introducing new theories, models, and methodologies for learning alongside robots while keeping the user and other stake-holders in the design process. Additionally, original technical research showcasing robot-centric systems, algorithms, and computational techniques designed specifically for real-time learner-robot interactions was encouraged. A key focus was to distinguish the unique aspects of learner-robot interaction from traditional human-robot interaction frameworks.

## 2 Research Topic formation

This Research Topic emerged from a series of workshops and research projects involving the editors, Dr Daniel Tozadore, Dr Jauwairia Nasir, A/Prof. Wafa Johal, and A/Prof Michelle M. Neumann. Individually, we were captivated by the opportunities and challenges of integrating robots into education, and collectively, we shared a deep enthusiasm for their potential to revolutionize social robotics in meaningful ways.

## 3 Contents of the Research Topic

### 3.1 Robots as tutors

The paper *Time-dependant Bayesian knowledge tracing—Robots that model user skills over time*, from Salomons and Scassellati, focuses on user skill modeling for Intelligent Tutoring Systems (ITS) and robotic tutors, particularly for complex tasks like programming and engineering. It introduces Time-Dependent Bayesian Knowledge Tracing (TD-BKT), an algorithm that continuously tracks user skills and provides more accurate teaching interventions. A user study demonstrated that a robot using TD-BKT effectively taught electronic circuit tasks, leading to significant skill improvements among participants.

Personalized learning is key to improving student engagement, yet traditional adaptive tutoring systems rely heavily on digital platforms and lack real-world social interaction. Socially assistive robots offer a promising alternative by integrating AI-driven assessments with real-time embodied social interactions. Maaz et al. present a tutor robot that personalizes education using facial expression analysis, subjective feedback, and performance metrics to classify students into tailored learning categories. The system employs the XGBoost algorithm to predict proficiency levels with high accuracy and dynamically adjusted learning content. The results show that the students using the AI-powered robotic tutor demonstrated significant improvement in learning outcomes compared to a control group that interacted with a human teacher.

### 3.2 Robot-mediated interactions

Educational social robots typically focus on domain knowledge, which is challenging as it requires either autonomous learning or frequent reprogramming. An alternative approach is designing robots that understand student behaviors linked to learning, rather than domain knowledge. In their paper, titled *Social robots as skilled ignorant peers for supporting learning*, Nasir et al. introduce *skilled ignorant peer* robots, which foster *productive engagement* using behavioral insights rather than domain expertise and formally investigate the relationship between (i) the robot interventions and students’ engagement, and (ii) students’ engagement and learning. A user study with 136 students compared two versions of such robots in real-time: *Harry* (focused on what to suggest) and *Hermione* (also considered when and why to intervene). While both robots led to similar learning gains, Hermione’s well-timed interventions were deemed more useful and indeed influenced students productive engagement state, highlighting the importance of timing in educational robot interactions.

In their paper titled *Fostering children’s creativity through LLM-driven storytelling with a social robot*, Elgarf et al. explore how a social robot could be used to stimulate children’s creativity by manipulating the level of creativity of a co-storyteller social robot. Through two user studies, with a total of 141 children, authors show that social robots prototyping creative behaviour can help children be more fluent and detailed in their idea generation. They demonstrate how LLMs can be used to scaffold the way children collaborate with social robots in a learning context.

### 3.3 Robots and stakeholders

Unlocking the full potential of telepresence robots for teaching in remote and local classroom environments is essential due to the changing nature of education and learning. Telepresence robots do not fully live up to the expectation of teachers and designers for supporting robot-mediated learning. In the paper *Robots for learning: An exploration of teacher roles, perceptions, and challenges in robot-mediated learning* Ahumada-Newhart et al. explore teachers’ experiences and perceptions of using telepresence robots for robot-mediated learning and provides valuable insights for best practice and highlights recommendations for technology designers.

Similarly, in their paper *Multiuser design of an architecture for social robots in education: teachers, students, and researchers perspectives*, Tozadore and Romero present the findings of an Interactive Design study exploring the challenges of integrating social assistive robots in classrooms, addressing both technical limitations and human factors such as teacher workload and stakeholder communication. It builds teachers and researchers experiences on the robotic architecture R-CASTLE, developed through extensive assessments with teachers and students, featuring an intuitive graphical interface designed for educators. Findings reveal that teachers can effortlessly incorporate their daily activities into the system without prior experience with social robots, demonstrating its usability and potential for classroom adoption.

### 3.4 Special education

Social robots have a wide application in special education as well. In the paper entitled *Managing social-educational robotics for students with autism spectrum disorder through business model canvas and customer discovery*, Arora et al. investigate the role of social-educational robotics, particularly humanoid robots like NAO, in supporting ASD students through social and collaborative interactions. It introduces a triad framework involving ASD students, teachers, and social robots while utilizing the Business Model Canvas (BMC) for robot design and curriculum development. Through customer discovery interviews, the study bridges academic research and industry, resulting in eight core propositions that enhance learning environments for ASD students and prepare them for future educational and workforce opportunities.

To assist the therapists in robot assisted therapy, this study by Stolarz et al. explores increasing the autonomy of robots by developing a learning-based behavior model using reinforcement learning. The authors tested different reward functions to optimize user engagement and activity performance and what they demonstrate is that strategies like policy transfer and learning from expert feedback help improve the learning process. A small-scale study with six users suggests that learning from guidance yields the most effective policies.

## 4 Conclusion

Advancements in the field of educational robotics follow the natural trajectory of technological development by proposing new algorithms for robot behavior regulation, human modeling to understand students’ cognitive states for robot mediation, and machine learning techniques to equip robots with convincing social behaviors. More interestingly, these advancements also consider external factors in their design, such as stakeholders’ opinions, students’ feedback and limitations, as well as business interests.

Finally, while the first editorial explores the broader role of robots in education, emphasizing stakeholder perspectives, social-emotional concerns, and diverse learning contexts, the second edition of the Research Topic leans towards AI-driven personalization, adaptive learning, and industry applications. Together, they highlight the evolution of educational robotics towards data-driven, personalized learning experiences and highlight several unique and important facets of robots for education.

